# Wear Resistance Improvement of Cemented Tungsten Carbide Deep-Hole Drills after Ion Implantation

**DOI:** 10.3390/ma14020239

**Published:** 2021-01-06

**Authors:** Dmitrij Morozow, Marek Barlak, Zbigniew Werner, Marcin Pisarek, Piotr Konarski, Jerzy Zagórski, Mirosław Rucki, Leszek Chałko, Marek Łagodziński, Jakub Narojczyk, Zbigniew Krzysiak, Jacek Caban

**Affiliations:** 1 Faculty of Mechanical Engineering, Kazimierz Pulaski University of Technology and Humanities in Radom, Stasieckiego 54, 26-600 Radom, Poland; d.morozow@uthrad.pl (D.M.); leszek.chalko@uthrad.pl (L.C.); 2 Plasma/Ion Beam Technology Division, National Centre for Nuclear Research Świerk, 7 Sołtana St., 05-400 Otwock, Poland; marek.barlak@ncbj.gov.pl (M.B.); zbigniew.werner@ncbj.gov.pl (Z.W.); jerzy.zagorski@ncbj.gov.pl (J.Z.); 3 Institute of Physical Chemistry, Polish Academy of Sciences, 44/52 Kasprzaka St., 01-224 Warsaw, Poland; mpisarek@ichf.edu.pl; 4 Vacuum Technique Laboratory, Łukasiewicz Research Network—Tele and Radio Research Institute, 11 Ratuszowa, 03-450 Warsaw, Poland; piotr.konarski@itr.lukasiewicz.gov.pl; 5 Zakład Mechaniki Maszyn BUKPOL Łagodziński Sp.j., Bukowiec, 96 Dolna, 95-006 Brójce, Poland; bukpol@bukpol.pl; 6 Institute of Molecular Physics, Polish Academy of Sciences, Smoluchowskiego 17, 60-179 Poznań, Poland; narojczyk@ifmpan.poznan.pl; 7 Faculty of Production Engineering, University of Life Sciences in Lublin, Głęboka 28, 20-612 Lublin, Poland; 8 Faculty of Mechanical Engineering, Lublin University of Technology, Nadbystrzycka 36, 20-618 Lublin, Poland; j.caban@pollub.pl

**Keywords:** deep-hole drilling, tools, guide pads, ion implantation, cemented tungsten carbide

## Abstract

The paper is dedicated to the life prolongation of the tools designed for deep-hole drilling. Among available methods, an ion implantation process was used to improve the durability of tungsten carbide (WC)-Co guide pads. Nitrogen fluencies of 3 × 10^17^ cm^−2^, 4 × 10^17^ cm^−2^ and 5 × 10^17^ cm^−2^ were applied, and scanning electron microscope (SEM) observations, energy dispersive spectroscopy (EDS) analyses, X-ray photoelectron spectroscopy (XPS) and Secondary Ion Mass Spectrometry (SIMS) measurements were performed for both nonimplanted and implanted tools. The durability tests of nonimplanted and the modified tools were performed in industrial conditions. The durability of implanted guide pads was above 2.5 times greater than nonimplanted ones in the best case, presumably due to the presence of a carbon-rich layer and extremely hard tungsten nitrides. The achieved effect may be attributed to the dissociation of tungsten carbide phase and to the lubrication effect. The latter was due to the presence of pure carbon layer with a thickness of a few dozen nanometers. Notably, this layer was formed at a temperature of 200 °C, much smaller than in previously reported research, which makes the findings even more valuable from economic and environmental perspectives.

## 1. Introduction

The cemented tungsten carbides, patented in 1923 in England and in the USA, are typically composed of a hard tungsten carbide (WC) phase held together by a soft ductile binder phase, usually cobalt [1]. Because of a combination of attractive features, such as strength, hardness, fracture toughness, refractoriness, stiffness, resistance to compressive deformation and wear resistance even at higher temperatures, WC-Co tools are widely used for metal cutting, wood machining, rock drilling, etc. However, the durability of tools is frequently not sufficient, which is why ion implantation was applied to improve their mechanical properties [2,3]. At present, more than 90% of all cemented carbide inserts underwent coating with chemical vapor deposition (CVD), physical vapor deposition (PVD) or a combination of both [4].

Numerous studies suggest that WC-Co damages can be interpreted as a continuous wear of the carbide during work of tools (or during mechanical tests) which leads to the formation of cracks in the material [5]. A variety of mechanisms have been proposed to describe the destructive phenomena in WC-Co, depending on the conditions of the work or laboratory tests [6,7]. Most of the mechanisms are correlated with each other [8], but in general, three levels may be emphasized—namely, WC grain-scale level, binder-scale level, and composite-scale level. A special case of the wear of WC-Co material is the corrosive wear, which may always occur in almost any environment, as several reports suggest [9,10]. This is due to the fact that the cobalt binder is the most susceptible to corrosion (selective binder leaching) in acid and neutral media, while WC-Co grains show active dissolution in alkaline media, in which the Co metallic binder passivates. Both metallic binders and ceramic particles are also damaged in dry environments. The character and the speed of the oxidation in air are closely related to temperature—namely, at about 400 °C, slight oxidation was observed, between 400 and 500 °C selective oxidation of Co binder appeared, and above 500 °C, simultaneous oxidation of the binder and WC phase took place [11,12]. Additionally, temperature can affect the binder properties, so that a temperature dependent fatigue effect takes place at temperatures between 25 and 900 °C, as reported in [13]. This effect can be ascribed to phase transformation from the face centered cubic (FCC) phase at low temperatures to the more brittle and hard hexagonal close packed (HCP) phase.

In order to enhance performance and durability of the WC-Co tools, some properties may be formed at the early stage of material preparation through selection of chemical composition, regulation of particles size, etc. In the case of ready-made tools, some modifications are still possible either by additional layers or by the introduction of additional elements by using surface engineering methods such as ion implantation [14] and, in particular, PVD or CVD [15,16], which leads to improved tribological behavior [17,18,19]. Important work was performed by Wang et al. [20] to help understand the effect of ion implantation on material lattice structures, mechanical properties and the nanometric cutting process of normal and implanted WCs. However, in the case of deep-hole drills, precise dimensions in the processed parts pose certain limitations on surface engineering applicability. It was proved, however, that ion implantation is a method that does not impact on the shape of tools, but functionalizes the surface properties [21]. It is a relatively cheap method for tool improvement, in which the process temperature can be performed even below 100 °C [22]. The modified region is not a layer, so no problem occurs with adhesion and a change of dimensions. Depending on the ion fluency (dose), this method leads to a hardness increase and microstructure changes in the surface layer, resulting in an increased wear resistance [23], decrease in the friction coefficient and decrease in the cutting force magnitude during machining of the Ti-6Al-4V alloy [24].

Previously, some studies were conducted on the ion implantation of the wood cutting tools and their models [25,26]. These studies demonstrated improvement of the average tool’s durability two times and more, and, among strengthening mechanisms, important factors were the appearance of the carbon-reach layer, formation of nitrides and interstitial nitrogen. In the present study, we focused on deep-hole drilling tools. This technology is commonly used in the aerospace [27] and automobile industries, as well as in the high-tech industries [28]. While this technology produces high-quality holes, a higher quality is pursued, so many studies are performed in order to improve the processing quality [29]. Coordination of the guide pads, cutting edges and machining parameters can produce exclusive surface features [30], but there is need to monitor fracture-related damage of both the workpiece and machine tool, and to perform recondition of the tool suitability for further use [31]. A cutting tool wear may be of a mechanical, adhesive, chemical and thermal nature [32,33,34,35,36,37]. Geometric changes are caused by friction related to the loss of the edge material and the changing local properties by plastic deformations, high temperatures and the chemical effect of the interacting medium [38]. Among various types of these tools, solid drill heads are frequently used. In these heads, the position of an external insert is fixed using cobalt-tungsten carbide guide pads. Their purpose is to burnish the processed surface of the prepared hole and to preserve coaxiality of the hole and the drill axis. This small part underwent investigations because it plays an important role during drilling and additionally protects the several thousand euros worth of solid drill heads from destruction. It was also reported that ultrasonic assistance was found to be more effective at high-depth ratio drilling of aluminum alloys [39].

Drill life improvement has been a concern of many researchers for many years [40,41]. Even though ion implantation technology has been used for improvement of many tools, its impact on the durability of the guide pads was never addressed properly. Commercial solutions for enhancement of the properties of this type of tools are usually associated with the TiN coatings, which have some shortcomings, such as dimensional change of the tool or layer delamination during the work. Ion implantation processes can be a good alternative for this sort of modification. In the following sections are presented the experimental conditions and results of a nitrogen ion implantation in WC-Co guide pads, which plays an essential role in the operational properties of deep drill cutting heads.

## 2. Materials and Methods

**Figure 1:**
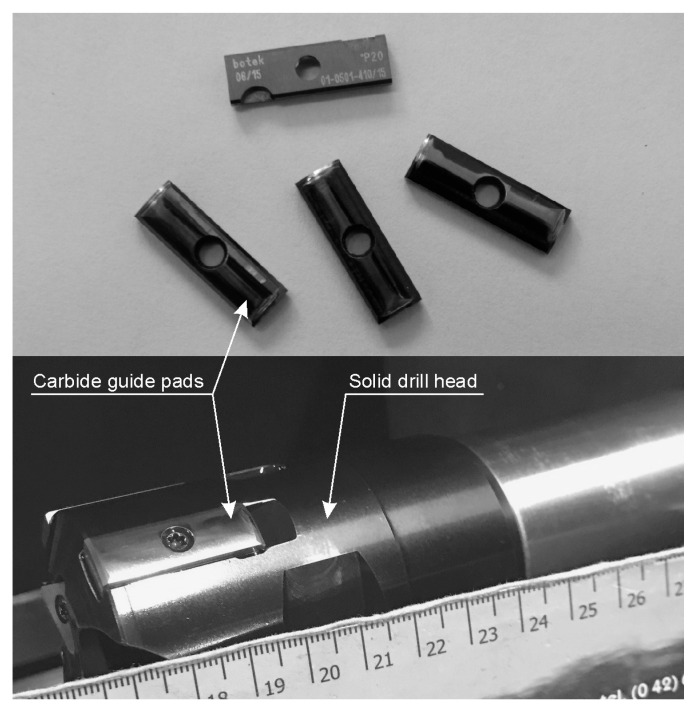
Carbide guide pads (**upper**) and solid drill head (**lower**).

The experiments were performed using available uncoated 01-0501-410/15 type WC-Co carbide guide pads, manufactured by Botek Präzisions-Bohrtechnik Gmbh company (Riederich, Germany). They are presented in Figure 1 together with the solid drill head equipped with the guide pads.

Before processing, the guide pads were washed in high-purity acetone under ultrasonic agitation. Next, their surfaces were implanted with nitrogen ions, using the semi-industrial ion implanter with a non-mass-separated ion beam. Nitrogen of 99.9% purity was used as the source of the implanted ions, based on initial studies [42]. The ion implantation of nitrogen was provided by two kinds of ions, i.e., N_2_^+^ and N^+^, in the ratio ca. 1:1. The implanted fluencies were 3 × 10^17^, 4 × 10^17^ and 5 × 10^17^ cm^−2^, respectively. Ions were implanted at a 60 kV acceleration voltage. The vacuum in the implanter working chamber was at a level of 2–4 × 10^−4^ Pa. The sample temperature did not exceed 200 °C. Afterwards, both nonimplanted and implanted guide pads underwent similar observations, comparative analyses and tests with apparatus mentioned in the following paragraphs.

The microstructure and the density of the guide pad material, its content and the distribution of the main compounds, oxygen and nitrogen, in the surface layer of several hundred nanometers thick, were examined with the use of a Zeiss EVO^®^ (Oberkochen, Germany) MA10 scanning electron microscope (SEM). It was equipped with an EDX Bruker XFlash Detector 5010 (Bruker Corp., Billerica, MA, USA) energy dispersive spectroscopy (EDS) system and dedicated Quantax 200, Esprit 1.9 code. The SEM observations were performed with magnifications of ×1000, ×5000 and ×10,000 and with the accelerating voltage of 20 kV, using Secondary Electron (SE, observations of cross-section) and Back-scattered Electron (BSE, observation of surface) detectors. The EDS investigations of microstructures of guide pad materials were made for the cross-section concerning chemical composition and for the surface concerning nitrogen content. Here, the magnifications of ×1000, ×5000 and ×10,000, and an accelerating voltage of 20 kV were also applied. In tungsten carbide, the range of electrons determined by Suspre code [43] and Quantax code for a depth of ca. 0.7 µm was 20 keV.

X-ray photoelectron spectroscopy (XPS) was used to analyze the chemical composition of the working surface of the guide pads and the nature of the chemical bonding on the surface modified by ion implantation. The surfaces before and after the implantation process were examined using a photoelectron spectroscope Microlab 350-Thermo Electron (Thermo Scientific, Waltham, MA, USA). The chemical states of surface species were identified using the high-energy resolution spherical sector analyzer of the spectrometer (SSA). XPS spectra were excited using Al*K_α_* (hν = 1486.6 eV) radiation as a source. The high-resolution W, C, O, Co and N spectra were recorded using 40 eV pass energy with a step of 0.1 eV. A Shirley background subtraction was made to obtain the XPS signal intensity. The peaks were fitted using an asymmetric Gaussian/Lorentzian (G/L = 35%) mixed function. The measured binding energies were corrected in reference to the energy of a C 1s peak at 284.8 eV originating from a C-C bond. Avantage-based data system software (Version 5.97) was used for data processing.

The depth profiles of W, C, Co, O and implanted N were determined using the Secondary Ion Mass Spectrometry (SIMS) analysis. SIMS analysis was performed on an SAJW-05 analyzer [44] equipped with a QMA-410 Balzers quadrupole mass spectrometer and 06-350E Physical Electronics ion gun. Ar^+^ (5 keV) was used as a primary ion beam. The incidence angle was 45°. The beam was scanned over a 1.6 × 1.6 mm^2^ area and the analysis encompassed 15% of the scanned area. The resulting sputtering rate for WC-Co substrates was 3.9 nm/min.

The microhardness measurements of the nonimplanted guide pads was performed by the Vickers method using Wilson Wolpert 401 MWD microhardness tester (Buehler, Lake Bluff, IL, USA). The instrument load was 0.2 N.

To evaluate the friction conditions on the working surface of the guide pads, tribological tests using Amsler apparatus were performed. For the tests, system with the guide pad and rotating counter-sample with a 53 mm diameter without greasing in an ambient atmosphere was applied. The glide velocity was v = 0.83 m/s, the load was F = 25 N and the friction distance was Lt = 2500 m. Friction torque Mt was recorded during the tests.

**Figure 2:**
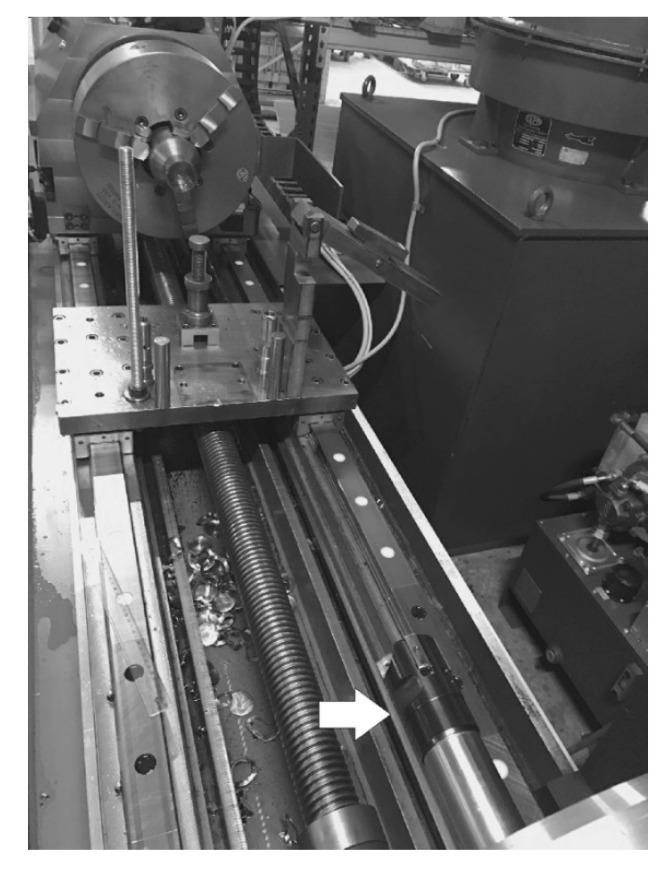
The view of the fragment of the TUC 40 lathe system used in the tests. The lying drill head is marked by the arrow.

To verify the effectiveness of nitrogen ion implantation on the working surface of guide pads in deep drill cutting heads, technological tests were performed in industrial conditions using TUC 40 lathe in the BUKPOL workshop. A fragment of the test apparatus is shown in Figure 2. The guide pad durability was estimated from the number of holes drilled to a wear limit determined by excessive quality changes in the holes, as regards the diameter and roughness of the surface.

The tests were performed at the following conditions: The processed material was 42CrMo4 steel with 28 HRC hardness. The length of the workpiece was 260 mm and its diameter was 54.92 mm. The cutting speed v_c_ = 50 m/min and the feed rate was f_n_ = 0.05 mm/rev. The intended diameter of the drilled hole was d_0_ = 16.02 mm. The cooling was performed using mineral oil with a flow of 70 l/min. To assess the drill durability, the obtained hole accuracy was measured. Since the hole had to be 16.02 mm in diameter, it was assumed that the tool should be considered worn out when the obtained diameter is 16.00 mm. Moreover, load on the spindle was measured since the increased load correlated with the drilled hole quality. The inner surface of the holes underwent visual inspection in order to identify the effect of cutting tool wear in form of scratches.

## 3. Results and Discussion

**Figure 3:**
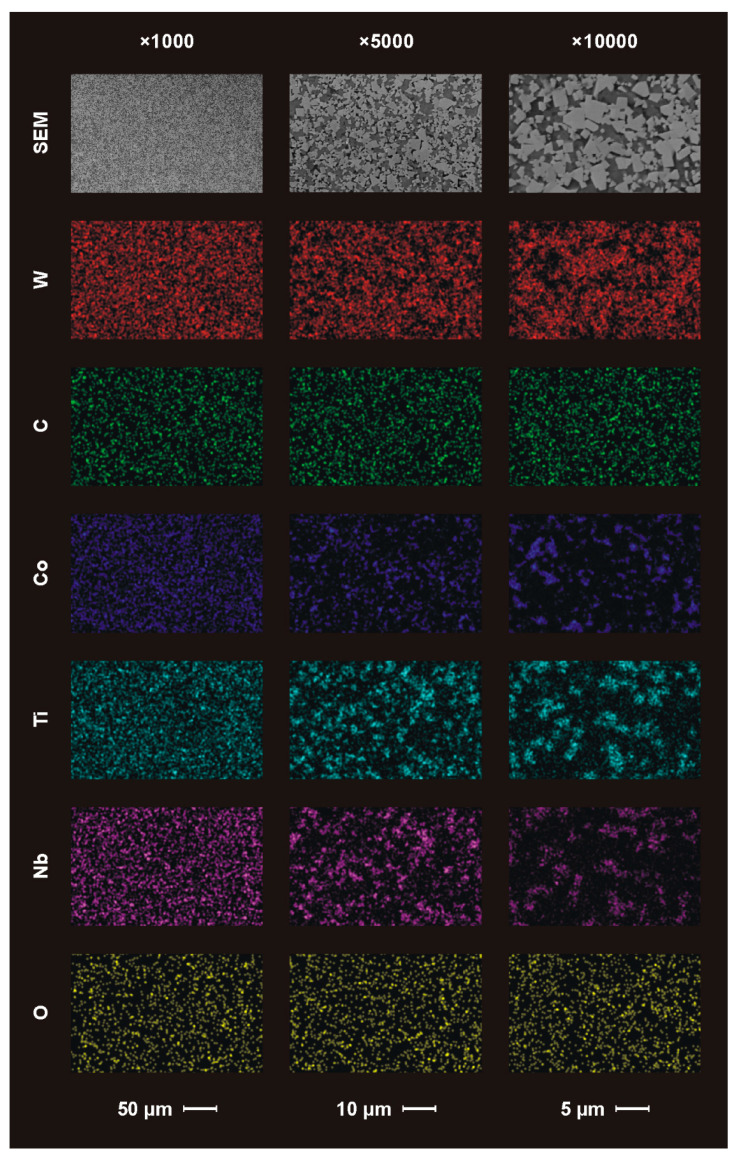
Scanning electron microscope (SEM) images and energy dispersive spectroscopy (EDS) maps of chemical composition of the nonimplanted guide pads’ surfaces.

The microstructure (top view denoted SEM) of the nonimplanted guide pads material and the chemical distribution of the elements (EDS mapping) are presented in Figure 3. The SEM images were recorded at magnifications of ×1000, ×5000 and ×10,000.

The SEM images (upper part of the Figure 3) show the light WC grains distributed in the dark Co binder area. They are distinguishable especially for magnifications of ×5000 and ×10,000. The grain size is few micrometers, though submicron grains are seen too. The grains’ ceramic particles have typical “sharp” shapes.

The EDS maps are presented in the second to last row of the Figure 3. The registered signal from the tungsten well correlates with the WC grains seen as SEM images, unlike the signal from carbon. The latter occurs both in the reinforcement and in the binder of the WC–Co composite. Presumably, the carbon-rich layer is the result of previous technological processes of the guide pad fabrication.

The EDS signal of cobalt correlates with the dark area at the SEM images of the composites. The binder phase is homogenously distributed between WC ceramic grains. The signal of the additional ingredients of the binder, i.e., titanium and niobium, correlates with the signal from cobalt, but this correlation is not uniform on the whole surface. In some places, Ti and Nb signals are stronger than the Co signal. Carbides of Ti and Nb were added to WC–Co composite to prevent deterioration of the tools’ properties at high cutting speeds and elevated temperatures [45].

The signal from oxygen originating from the atmosphere is registered on the entire surface, such as that of carbon, but its distribution is less homogeneous.

Besides W and C, the chemical compositions of the original materials, as determined by the EDS method, were, on average, ca.: 11.8% of Co, 4.2% of Ti and 4.4% of Nb. Additionally, about 1.1% of O was detected via net-count ratios. The density of this material was 15.44 g/cm^3^.

**Figure 4:**
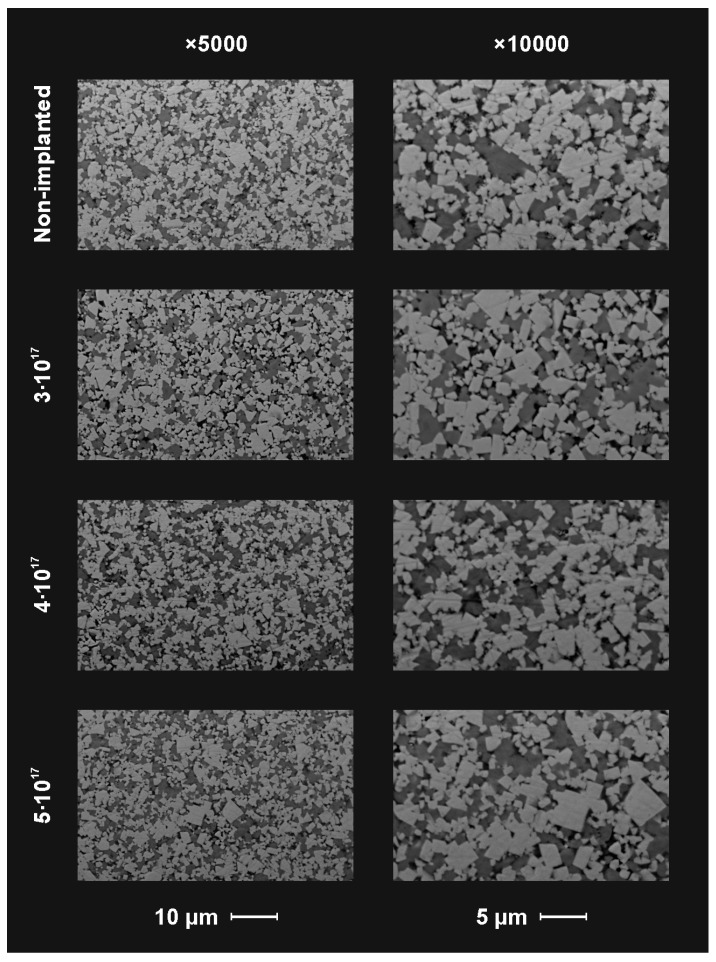
SEM observations of the nonimplanted and implanted with 3 fluencies on the surface of the guide pad material.

After surface characterization of nonimplanted materials by SEM and EDS methods, the guide pads were functionalized by the nitrogen ion implantation process. The content of the implanted nitrogen in the layer with a thickness of several hundred nanometers, in weight percentages, was on average about: 1.3% for 3 × 10^17^ cm^−2^, 1.7% for 4 × 10^17^ cm^−2^ and 2.1% for 5 × 10^17^ cm^−2^ or in atomic percentages, about: 5.5% for 3 × 10^17^ cm^−2^, 6.5% for 4 × 10^17^ cm^−2^ and 7.5% for 5 × 10^17^ cm^−2^. SEM images of the surfaces after ion implantation with three fluencies are shown in Figure 4.

From the microscopic observation of the implanted and nonimplanted guide pads, it can be concluded that ion implantation did not change the surface layer structure pattern distinguishably at the microscale. Hence, the applied method of surface engineering had no impact on the shape of tools either.

**Figure 5:**
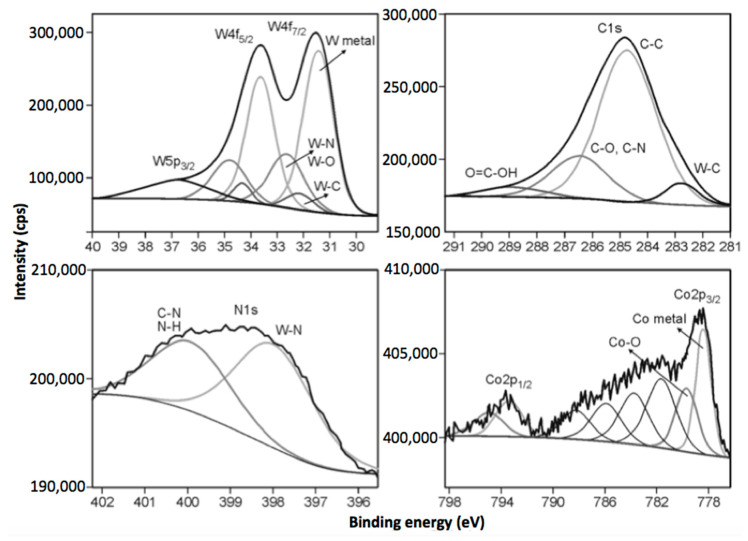
High-resolution XPS spectra of W4f (**upper left**), C1s (**upper right**), N1s (**lower left**) and Co2p (**lower right**) of the nitrogen implanted guide pads (3 × 10^17^ cm^−2^).

For determination of surface chemistry of the nonimplanted and implanted guide pad materials, the XPS (X-ray Photoelectron Spectroscopy) investigations were performed. Accurate measurements of the XPS peak positions can inform on the chemistry of the analyzed elements [46]. The measured binding energies of the core electrons provided a large amount of information about the properties of the atoms in the molecules and bulk material. The results of XPS measurements are summarized in Table 1 and Figure 5. For all regions, the peaks are located at positions which correspond to the available reported data for tungsten nitrides, tungsten oxides, tungsten carbides, cobalt metal and cobalt oxides [47,48,49].

Apart from nitrogen implanted on the WC-Co samples and identified structurally as nitrides, there is also identifiable in the C1s spectra some nitrogen adsorbed in the surface, shown as C-NH_x_ in Table 1. Being a sort of contamination, this nitrogen does not affect durability of the ion implanted tools. The amount of structural nitrogen, which is crucial for enhanced properties, increased along with implantation dose.

The deconvolution of the W4f doublet is not straightforward due to the relative proximity of the binding energy positions of the metal phase, carbide, nitride and low valence oxides of tungsten, taking into account the fact that the kinetic energy resolution of the analyzer was 0.83 eV. For all tested materials, W4f peaks were resolved into the W4f_7/2_ and W4f_5/2_ doublet. The separation energy between these two peaks was 2.18 eV, keeping the ratio of those spectra line 4:3, respectively. It was usually possible to distinguish three different doublets for WC-Co samples: a main peak at 31.1 eV for nonimplanted sample and 31.6 (3 × 10^17^), 31.8 (4 × 10^17^) and 31.4 eV (5 × 10^17^) for implanted materials at higher binding energies. The main components at lower energies can be attributed to W-W bonds, tungsten carbides and probably tungsten nitrides [50]. The next components of the W4f peaks are related to the position of tungsten oxides—W^4+^ (32.8, 33.0, 32.5 eV)—and the last one can be attributed to nonstoichiometric W oxides (33.7, 34.0, 33.4 eV) [48]. The C1s and N1s high-resolution XPS spectra confirm the presence of carbide and nitride components in the tested materials. The carbide peaks at 282.7, 282.8 and 282.9 eV, which correspond to chemical bonding between W and C, were found. The characteristic peak positions for nitrides were also identified at 398.0 (3 × 10^17^), 398.3 (4 × 10^17^), 397.9 eV (5 × 10^17^), respectively [47,50]. Furthermore, additional shifts in nitrogen peaks toward higher binding energies were observed, suggesting that some nitrogen is chemisorbed to the surface of the implanted samples. The deconvolution of Co2p spectra revealed the existence of cobalt in two various chemical states. The main signals at 778.5, 778.6 and 778.7 can be attributed to metallic state (Co^0^). The second peaks located at 779.7, 779.8 and 780.0 can be assigned to Co oxides (Co^2+^, Co^3+^—Co_3_O_4_).

The XPS results also reveal that the concentration of nitrides after implantation changed from 2.1 to 3.6 at. %, depending on the conditions of the process, which has an impact on the improvement of the mechanical properties of the modified surface of the plates.

**Figure 6:**
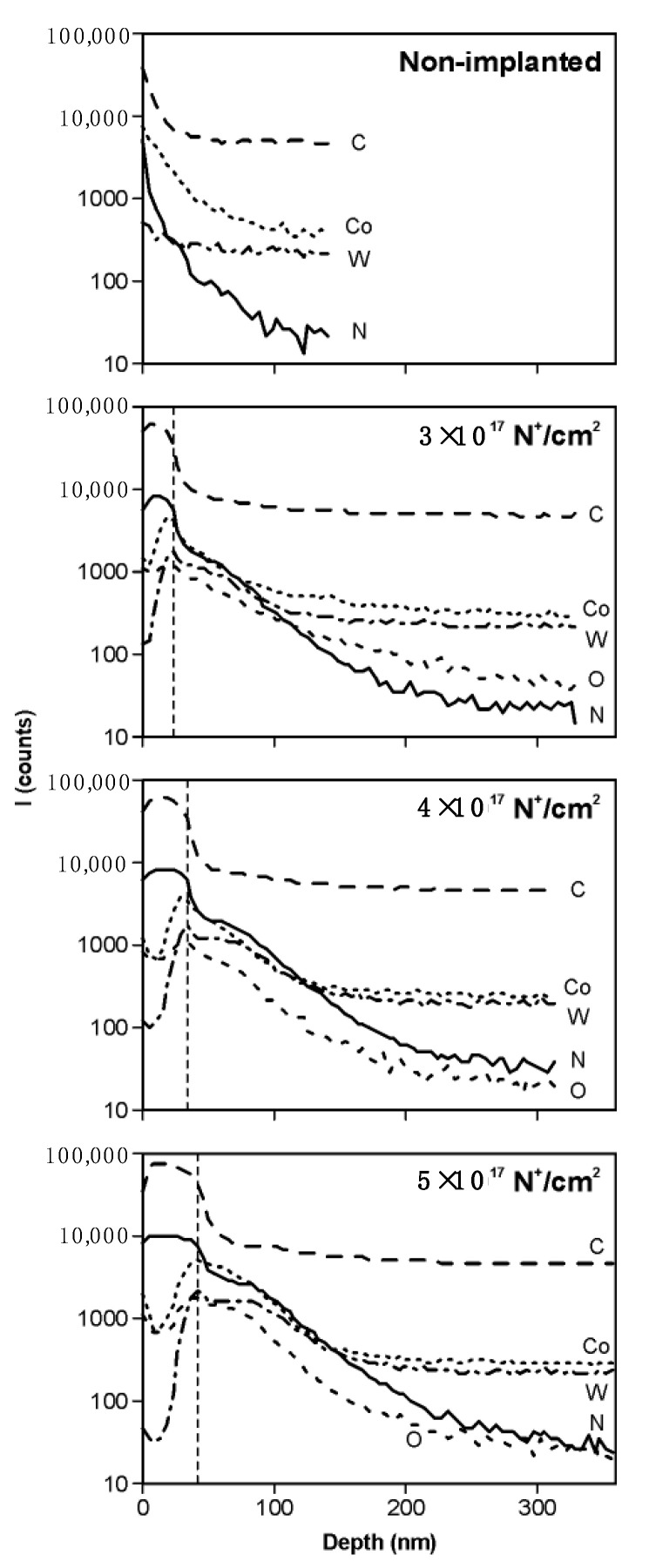
Secondary Ion Mass Spectrometry (SIMS) depth profile analyses of the nonimplanted sample and samples implanted with nitrogen.

Figure 6 shows the result of SIMS depth profile analysis of the elements W, C and Co for nonimplanted sample and for the samples implanted with nitrogen at three fluencies: 3 × 10^17^, 4 × 10^17^ and 5 × 10^17^ cm^−2^. C^+^ and CH_2_^+^ ion plots show that the surface of the samples after or during ion implantation process is covered with a carbon layer with a thickness depending on the implantation fluency and is 20, 30 and 40 nm, respectively. During the profile analysis, secondary ion currents of the main components of the substrate were recorded. These are: C^+^ (m/z = 12D), Co^+^ (59D) and W^+^ (186D). Additionally, ions with m/z = 14D, representing the N^+^ implant ions and CH_2_^+^ molecular ions as well as O^+^ (16D) ions, were recorded.

The plots of m/z = 14D ion currents show that below the carbon layer there is a layer enriched with the implanted nitrogen. We assume that after sputtering of the carbon layer, the m/z = 14D secondary ions are mainly represented by N^+^ ions. The thickness of this implanted layer can be estimated as approximately 50 nm. Slight oxidation of this layer causes typical effect for SIMS analysis—i.e., ionization of the electropositive components of the substrates cobalt and tungsten is increased. As a result, we noted higher currents of Co^+^ and W^+^ secondary ions, as compared with those recorded at depths above approximately 200 nm.

**Figure 7:**
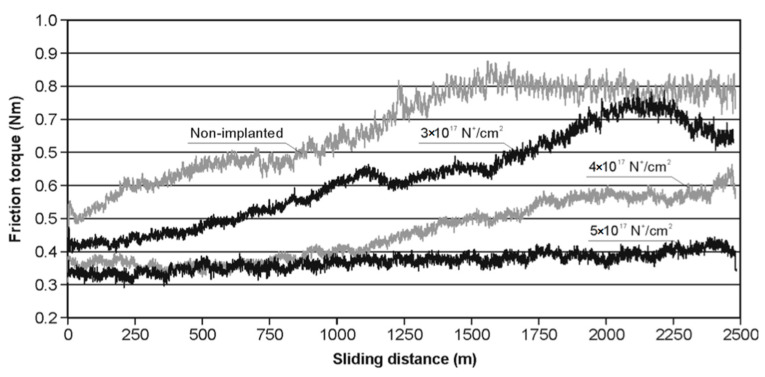
Friction torque as a function of friction distance for the guide pads.

The abovementioned characteristics had a direct impact on the mechanical properties, which is important from the practical perspective of industrial applications. Figure 7 presents a graph of friction torque versus sliding distance for the nonimplanted guide pads with microhardnesses HV_0.2_ of 1480 and for the implanted ones. It is notable that the higher the implanted ions’ fluency, the lower the values of friction torque and friction resistances. In the case of 5 × 10^17^ cm^−2^ fluency, the value of friction torque is almost linear for all friction distances, and the maximum variation is no more than about 30%. In other cases, the character of the friction torque graph is more difficult to assess and the variation changes were even as high as 100%.

**Figure 8:**
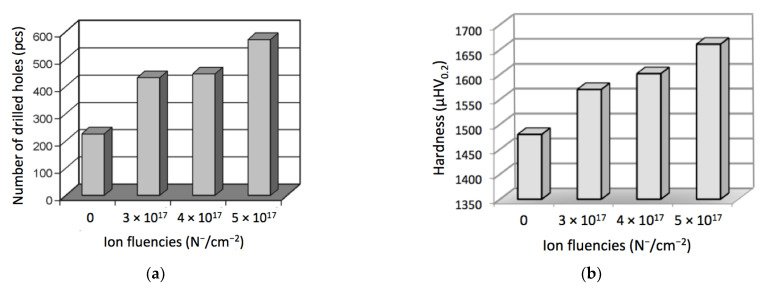
Effect of ion implantation on the durability and hardness of the WC-Co drills: (**a**) number of the holes drilled before the tool was worn out; (**b**) microhardness dependent on the ion fluency.

It is widely recognized that there is direct relationship between surface topography, manufacturing process and function of the produced part [51]. The surface morphology of the investigated guide pads after machining tests indicated a typical abrasive nature of the wear. The “two body abrasive wear” friction process was clearly present. Additionally, the presence of the hard tungsten nitrides and tungsten oxides could suggest the existence of “three body abrasive wear” friction process. The results of technological tests of the implanted and nonimplanted guide pads are shown in Figure 8a, where durability is represented by the number of holes drilled before the pad was worn out. Similarly, the effect of ion implantation on the microhardness is presented in Figure 8b.

Noteworthy, different fluencies did not have a proportional effect either on durability or on the microhardness of the cutting tools. Differences between nonimplanted and implanted tools’ lifetimes is almost twofold, while differences between the ones implanted with fluencies of 3 × 10^17^ cm^−2^ and 4 × 10^17^ cm^−2^ are quite small. In the future, the exact relation between fluency and properties of the carbide tools should be examined, but the results obtained so far demonstrate that the nitrogen ion implantation of guide pads is highly advantageous from the perspective of drill durability in the deep-hole drilling process. Increase in durability after ion implantation with a fluency of 5 × 10^17^ cm^−2^ provided 2.5-fold longer drill lifetime.

Evidently, the performance improvement of the pads can be attributed to the reduction in the friction values of all implanted samples, caused by dissociation of the tungsten carbide phase. A layer of pure carbon with a thickness of a few dozen nanometers, as determined by the SIMS method, acted as a lubricating film, reducing the friction [52]. Additionally, extremely hard tungsten nitrides, synthesized from free tungsten and the implanted nitrogen, as determined by the XPS method [53] strengthened the implanted layer [54]. The reaction of the nitride synthesis was previously reported at a temperature of 400 °C [55], whereas in our studies, the temperature of the implanted samples did not exceed 200 °C. Thus, at least two mechanisms for extending tool life, namely, carbon lubrication and nitride strengthening, can be attributed to the increased durability of the nitrogen implanted WC-Co guide pads.

## 4. Conclusions

Results of technological tests in industrial conditions of guide pads in drilling heads for deep drills confirmed that nitrogen ion implantation of their working surfaces substantially increased tool durability. The guide pads implanted with fluencies of 3 × 10^17^ cm^−2^ and 4 × 10^17^ cm^−2^ were able to manufacture two times more holes than nonimplanted ones. For a fluency of 5 × 10^17^ cm^−2^, the durability increased even more (above 2.5 times). More exact dependence of the durability on ion implantation fluency should be investigated in future research.

Evidently, the performance improvement of the pads can be attributed to the reduction in the friction and respective wear in all implanted samples, caused by the dissociation of tungsten carbide phase. Laboratory analyses confirmed the presence of a layer of pure carbon with a thickness of a few dozen nanometers, which perhaps acted as a lubricating film and reduced friction forces. As follows from the analysis, larger doses of nitrogen ions formed a thicker carbon layer which maintained a reduced friction after subsequent nanolayers were worn out. Additional strengthening of the implanted surface layer was presumably caused by the extremely hard tungsten nitrides, synthesized from free tungsten and the implanted nitrogen. This effect was obtained at a temperature of 200 °C, much smaller than in previous reports. Thus, the structure of ion implanted material was not affected by high temperature, showing high mechanical characteristics. Additionally, the reduced temperature makes our finding even more valuable from economic and environmental perspectives. Additionally, titanium may strengthen the material structure through the formation of some nitrides, as indicated in other sources. In the future, research on its effect should be additionally examined.

## Figures and Tables

**Table 1:** W4f_7/2_, Co2p_3/2_, N1s and O1s binding energies evaluated from a deconvolution procedure of corrected X-ray photoelectron spectroscopy (XPS) spectra for Tungsten carbide (WC)-Co samples before and after implantation process.

Materials	Binding Energy (eV) High-Resolution Spectra					Chemical State
WC-Co nonimplanted	W4f_7/2_	Co2p_3/2_	N1s	O1s	C1s	
	31.1				282.9	W^0^, W-C (WC_x_)
	32.2				282.9	W in WC
		778.5				Co^0^
	32.8	779.7		530.6		W^4+^, Co^2+^-Co^3+^
WC-Co 3 × 10^17^	W4f_7/2_	Co2p_3/2_	N1s	O1s	C1s	
	31.6				282.8	W^0^, W-C (WC_x_), W-N (WN_x_)
			398.0			C-NH_x_
			400.2			Co^0^
	32.8	778.5				W^4+^, Co^2+^-Co^3+^
	33.7	779.7		530.5		W-O (WO_x_)
WC-Co 4 × 10^17^	W4f_7/2_	Co2p_3/2_	N1s	O1s	C1s	
	31.8					W^0^, W-N (WN_x_)
			398.3			C-NH_x_
			400.1			N-H (NH_x_)
			402.6			Co^0^
	33.0	778.7				W^4+^, Co^2+^-Co^3+^
	34.0	779.8		530.6		W-O (WO_x_)
WC-Co 5 × 10^17^	W4f_7/2_	Co2p_3/2_	N1s	O1s	C1s	
	31.4				282.7	W^0^, W-C (WC_x_), W-N (WN_x_)
			397.9			C-NH_x_
			400.1			Co^0^
	32.5	778.6				W^4+^, Co^2+^-Co^3+^
	33.4	780.0		530.3		W-O (WO_x_)

## Data Availability

Data available on request due to privacy restrictions.
